# Containing the Damage of Unfolded Proteins

**DOI:** 10.1371/journal.pbio.0040442

**Published:** 2006-11-28

**Authors:** Liza Gross

The first stop for proteins bound for secretion from the cell or tethered to its membrane is the endoplasmic reticulum (ER). After secretory gene transcripts are translated into amino acid chains by ribosomes on the ER, they enter the ER’s labyrinthine network of membranes as extended polypeptide chains. Once the polypeptides are inside, ER enzymes assemble, modify, and fold them into their proper 3-D conformation in preparation for the next stop on the secretory pathway.

When protein-folding demand exceeds ER capacity and clogs up the system with incorrectly folded proteins, sensors on the ER membrane stimulate the “unfolded protein response” (UPR). This ER-to-nucleus signaling pathway controls a vast gene-expression program that adjusts ER processing capacity and restores homeostasis by adjusting the size of the ER compartment, enlisting protein-folding enzymes, regulating the entry of amino acid chains, and removing irreparably folded proteins through the ER-associated protein degradation pathway.

In a new study, Sebastián Bernales, Kent McDonald, and Peter Walter report a surprising link between another protein-degradation pathway, called autophagy, and the UPR. They show that as yeast cells expand their ER to accommodate increased demand, they also synthesize autophagosome-like bodies that sequester stacks of membrane from the expanded ER. This mechanism may allow the ER to keep the protein production line operating at a steady state even as misfolded proteins accumulate, and it may also control the size of this organelle.

To characterize the UPR’s effect on ER structure and size, Bernales et al. chemically induced the UPR in yeast cells. Electron microscopy analysis revealed a “massive expansion” of the ER in treated cells compared with untreated cells. In yeast, the primary UPR sensor, Ire1, triggers the UPR by promoting the synthesis of the transcriptional activator Hac1. When the authors directly activated Ire1’s downstream transcriptional targets without causing protein misfolding (by artificially activating Hac1 production), they saw a similar increase.

On closer inspection of UPR-induced cells, the authors were surprised to see an abundance of “autophagosome-like structures packed with tightly stacked membrane cisternae.” The structures were surrounded by double membranes like autophagosomes and were about the same size. The authors called them ER-containing autophagosomes (ERAs) after determining that their membrane contents came from the ER, based on the presence of ribosomes and other ER proteins and extensive membrane visualization analysis. Three hours after UPR induction, the majority of cells had proliferated ERs and about 20% of cells had ERAs. None of the ERA-containing cells had an expanded ER, suggesting that ERAs provide a means to downsize the ER to counteract expansion.

**Figure:**
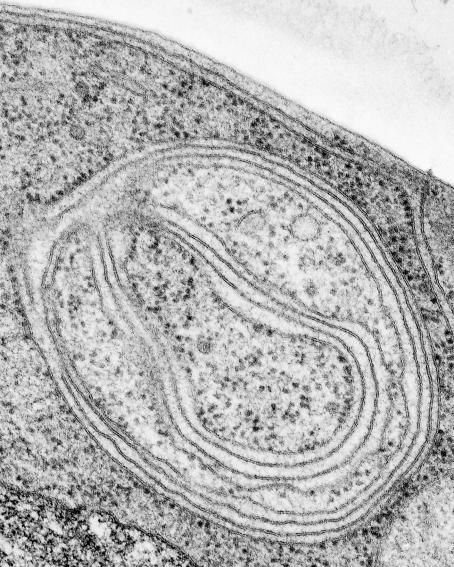
Cutting-edge electron-microscopy visualization techniques revealed that portions of both the internal membranes and the sequestering double-membrane envelope of the ER-containing autophagosome contain membrane-bound ribosomes.

Given the ERAs’ structural resemblance to autophagosomes, the authors reasoned that they may function similarly as well. In autophagy, cells recycle excess or worn out organelles (and sections of cytoplasm) by packaging them into autophagosomes that fuse with vesicles (vacuoles or lysosomes) equipped with protein-digesting acid hydrolases. Starvation induces macro-autophagy, which indiscriminately cannibalizes cytoplasm sections to release nutrients from degraded molecules. By contrast, ERAs are highly selective in sequestering ER membranes.

To investigate ERA function, the authors tracked the fate of an early component of autophagosome formation, Atg8, using a fluorescent version of the protein. In nitrogen-starved yeast, Atg8 concentrates in pre-autophagosomal structures (PASs) near the vacuole, where Atg8’s fluorescent domain is removed. It is thought that PASs stimulate the formation of autophagosomes, which then fuse with the vacuole and dump their contents. As expected, nitrogen starvation upregulated Atg8 (but not the UPR relay signal). Chemically induced UPR also increased Atg8 production, but did not cleave Atg8’s fluorescent domain, indicating that Atg8’s fate differs under these different physiological stresses. But more surprising, this result identifies the autophagy gene *ATG8* as a UPR target.

The authors go on to show that UPR induction produces a “vast proliferation” of Atg8-containing PASs, located near vacuoles or ERAs (when cells had them). Since no ERAs formed in cells lacking *ATG8*, the gene appears to play a part in ERA formation. *ATG8*, along with five other autophagy genes, is also required for cell growth under UPR-inducing conditions.

Altogether, these results establish a surprising link between autophagy and the UPR. Autophagy, the authors conclude, supplements the UPR by selectively “self-eating” excess ER to help cells weather the potentially fatal consequences of ER stress—in contrast to starvation-induced autophagy, which cannibalizes cellular contents for their metabolites. Intriguingly, autophagy genes can be enlisted by Ire1 but also independently of the Ire1-dependent UPR pathway, thus providing a rich platform for exploring alternative ER-to-nucleus signaling pathways in yeast. Future studies can begin to characterize the molecular mechanisms required for ERA formation and more fully explore the cellular functions of “ER-phagy.”

